# Gastric outlet obstruction possibly secondary to ulceration in a 2-year-old girl: a case report

**DOI:** 10.1186/1757-1626-2-8

**Published:** 2009-01-05

**Authors:** Manabu Okawada, Tadaharu Okazaki, Tsubasa Takahashi, Geoffrey J Lane, Atsuyuki Yamataka

**Affiliations:** 1 Department of Pediatric General and Urogenital Surgery, Juntendo University School of Medicine, 2-1-1 Hongo, Bunkyo-ku, Tokyo 113-8421, Japan

## Abstract

Gastric outlet obstruction due to ulceration is extremely rare in childhood. We report a case of gastric outlet obstruction possibly secondary to peptic ulceration and our surgical management. Our approach, without vagotomy or antrectomy, would appear to be a safe and effective.

## Background

Gastric outlet obstruction (GOO) may be caused by peptic ulceration, caustic ingestion, tumor, chronic granulomatous disease, or eosinophilic gastroenteritis [1-6], but peptic ulceration is extremely rare in childhood with an incidence of only 1 in 100,000 live births [1,2,7].

The introduction of histamine-2 (H_2_) receptor blockers, proton pump inhibitors (PPI), and treatment for *Helicobacter pylori *(*H pylori*) has revolutionized the non-surgical management of peptic ulcer disease, with most patients now being treated conservatively [8,9].

Herein, we present a case of severe GOO possibly secondary to peptic ulceration despite intravenous administration of H_2 _receptor blockers who required surgical intervention.

## Case presentation

**Figure 1:**
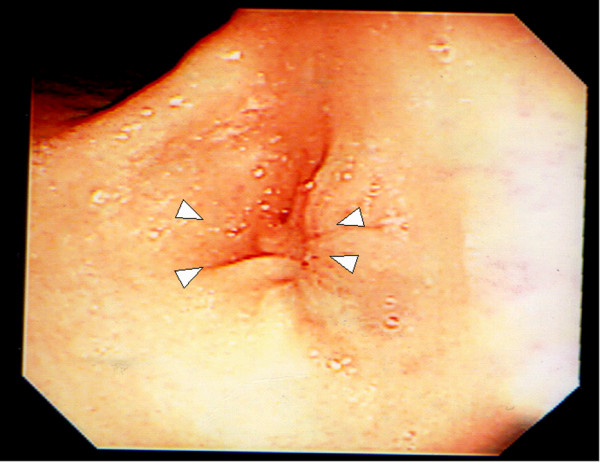
**Endoscopic finding before surgery**. Arrowheads indicate the strictured antrum with normal mucosa.

**Figure 2:**
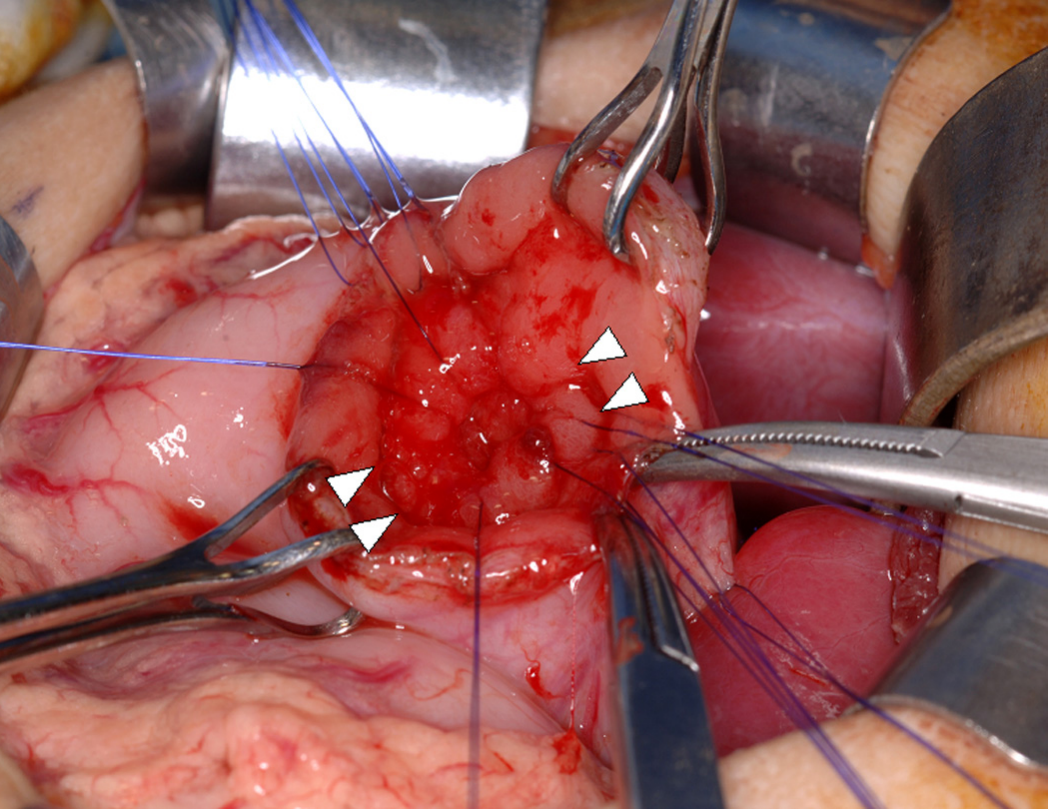
**Our operative procedure**. A mucosal incision was made circumferentially 5 mm proximal to the antral stricture (arrowheads: between inner and outer stay sutures) to allow the mucosa to be dissected free from underlying fibrotic scar tissue which was excised with great care not to perforate the gastric wall.

**Figure 3:**
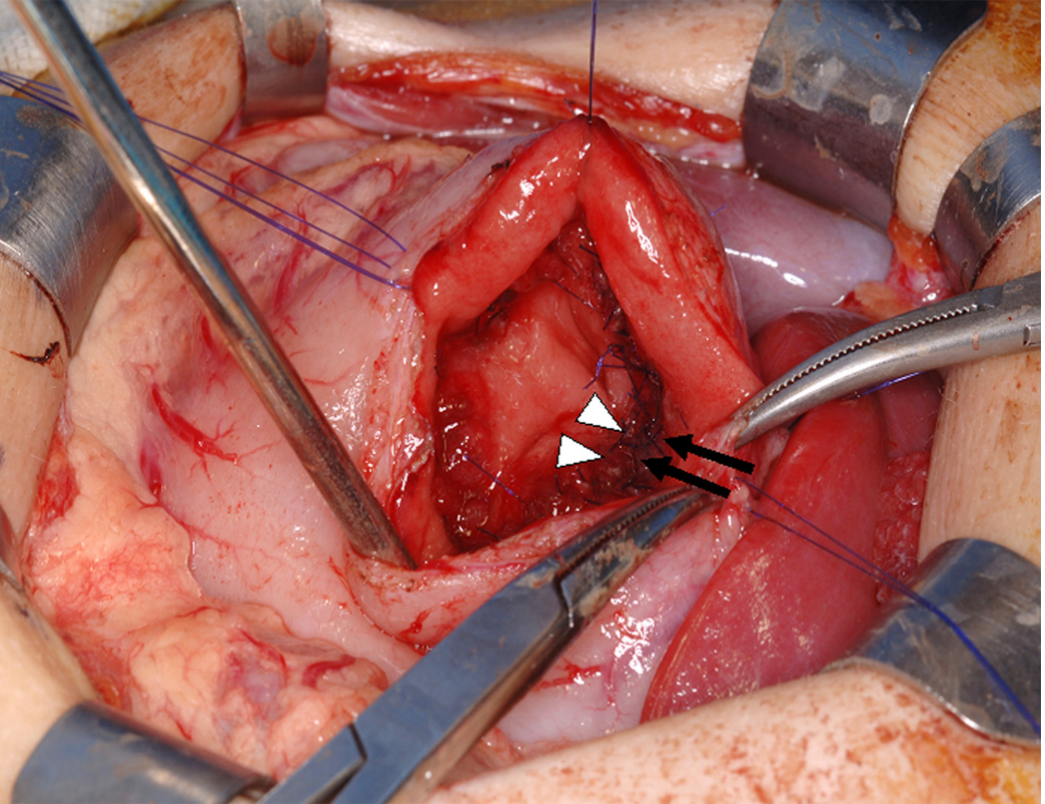
**procedure continued: **The mucosa, submucosa, and healthy muscle layers (arrows and arrowheads) were approximated.

**Figure 4:**
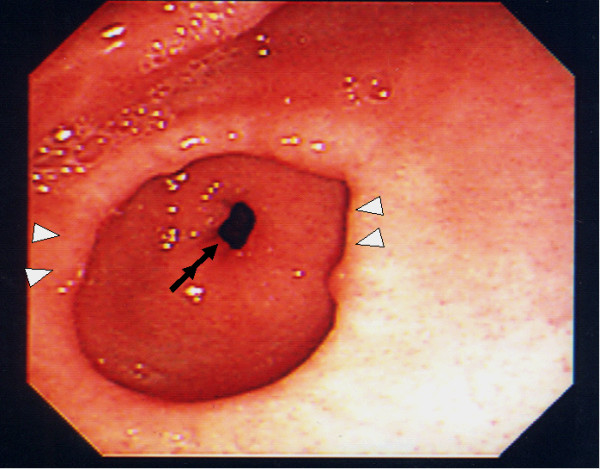
**Endoscopic finding 6 months after surgery**. No stricture is seen.(arrowheads: the expanded antrum, double arrows: pyloric ring).

A previously healthy 2-year-old girl contracted influenza and developed epigastric pain and tarry stools. H_2_-blockers, PPI and enterokinesis activators were commenced elsewhere. Two weeks later, she was referred to us for intractable vomiting and epigastric pain. There was no family history of peptic ulcer. Abdominal radiology was suggestive of pyloric obstruction but ultrasonography showed a normal pylorus. On barium meal, there was no passage of barium from the stomach to the duodenum and the stomach was distended with increased peristalsis. On endoscopy, the antrum was so narrowed circumferentially that the endoscope could not be passed through, but surprisingly, the mucosa was normal without scarring (Fig. 1). Serum gastrin was normal, and serology and biopsies for *H pylori *were negative. Imaging analyses and laparoscopic investigation revealed no obvious compression from surrounding organs. Endoscopic balloon dilatation was performed twice without improvement so surgery was planned. At laparotomy, gastrotomy was performed transversely over the antrum just proximal to the stricture and the lesion delivered through the incision (Fig. 2). The mucosa was incised circumferentially 5 mm proximal to the antral stricture and dissected free from underlying fibrotic scar tissue which was then excised with great care to not perforate the gastric wall. After excising the fibrotic tissue, the stricture was released and the antrum expanded nicely. The mucosa, submucosa, and healthy muscle layers were then approximated (Fig. 3). An endoscopic finding 6 months after surgery, no stricture is seen (Fig. 4).

## Discussion

GOO is extremely rare in children and its management is not established [8]. There are some reports of children with GOO secondary to peptic ulceration being treated successfully with vagotomy combined with pyloroplasty, anterectomy, or gastrojejunostomy [1,10,11] and Billroth I, Billroth II, or pyloroplasty have also been used to treat GOO with good results. In these reports, peptic ulceration did not recur and failure to thrive was not seen in any of these cases in childhood. However, anterectomy is relatively invasive in children, and gastrojejunostomy has a risk for peptic ulceration on the anastomotic site. Pyloroplasty may change the angle of the pyloric canal which may disrupt the smooth passage of stomach contents into the duodenum postoperatively. We experienced such complications in patients who had pyloroplasty elsewhere. Some authors have advocated balloon dilatation of the pylorus in adults, but its long-term efficacy has not been proven in children [12]. In adults, the short-term failure and long-term recurrence rates for balloon dilatation have been reported to be as high as 30–84% [13-15]. In our case, balloon dilatation was not effective, and we perform the procedure described here for GOO in children.

In the present case, we did not perform vagotomy because serum gastrin was normal and *H pylori *was negative. Surprisingly, the mucosa of the antral stricture was normal and no mucosal scar was identified on preoperative endoscopy or intraoperatively, although mucosal scarring is usually present after treatment of peptic ulceration according to other reports in the literature [1,13-15]. Our procedure is simple and does not cause any anatomic or physiologic changes.

We believe our procedure is suitable for patients with GOO secondary to peptic ulceration and should be considered as one of the indications for surgical intervention in children with GOO with normal serum gastrin.

## Abbreviations

GOO: Gastric outlet obstruction; H_2_: Histamine-2; PPI: Proton Pump Inhibitors; H pylori: Helicobacter Pylori

## Consent

Written informed consent was obtained from the patient for publication of this case report and accompanying images. A copy of the written consent is available for review by the Editor-in-Chief of this journal.

## Competing interests

The authors declare that they have no competing interests.

## Authors' contributions

MO did the literature search and wrote the case report and also obtained written consent. AY conceived the study and helped to draft the manuscript. TO, TT and GJL prepared the manuscript and helped in the literature search. All authors had gone through the final manuscript and approved it.
